# Lessons From Italy's and Sweden's Policies in Fighting COVID-19: The Contribution of Biomedical and Social Competences

**DOI:** 10.3389/fpubh.2020.563397

**Published:** 2020-09-22

**Authors:** Mirko Farina, Andrea Lavazza

**Affiliations:** ^1^Institute for Humanities and Social Sciences, Innopolis University, Innopolis, Russia; ^2^Institute of Philosophy, Saint Petersburg State University, Saint Petersburg, Russia; ^3^Department of Philosophy, King's College, London, United Kingdom; ^4^Centro Universitario Internazionale, Arezzo, Italy

**Keywords:** COVID-19, public health, expertise, scientific disagreement, ethics, science

## Abstract

We start (section The COVID-19 Pandemic and Italy's Response to It) by focusing on Italy's “tough” response to COVID-19 pandemic, which included total lockdown with very limited possibility of movement for over 60 million individuals. We analyse (section Sweden's Softer Approach) Sweden's softer approach, which is based on relatively lax measures and tends to safeguard fundamental constitutional rights. We problematise (section General Disagreement Among Experts: A Pressing Epistemic Problem) around the stalemate that arises as a consequence of the implementation of these different approaches, both epistemically grounded and equally justified, in the face of an unknown virus, in society. We point out that in some cases, like the one we discuss here, the epistemic justification that underlies scientific expertise is not enough to direct public debates and that politicians shouldn't exclusively focus on it. We claim that, especially in situations of emergency when experts disagree, decision makers ought to promote broad discussions, with attention to public reason as well as to constitutional rights, in the attempt to find a shared procedural and democratic agreement on how to act. On these grounds (section The Need of More Public Discourse in Fighting Covid-19) we call for an increase role of different types of expertise in public debates thus for the inclusion of ethicists, bioethicists, economists, psychologists, moral and legal philosophers in any scientific committee responsible for taking important decisions for public health, especially during situations like pandemics. Likewise, in the interest of public reason and representativeness, we also claim that it may be fruitful to bring in non-experts, or experts whose expertise is not based solely on “epistemic status,” but rather on either experience or political advocacy, of either the homeless, the immigrant, or other disenfranchised groups. This, in expanding the epistemic-expert pool, may also make it “more representative of society as a whole.”

## The COVID-19 Pandemic and Italy's Response to it

As of September 2020, SARS-CoV-2 -a coronavirus which likely originated in Wuhan, China- that causes COVID-19 - has been ravaging the world (almost 30 million people infected), causing the deaths of almost 1,000,000 people (at the time of writing)^1^.

The virus's etiology is still not-well understood; however, it is known that it propagates quickly among humans by close contact, air currents, by touching contaminated objects or through respiratory droplets produced when an infected patient coughs or sneezes^2^. The virus may cause, in its strongest manifestations, acute respiratory infections that lead to the death of the individual that contracted the virus [estimated mortality rate was 3.4% as of March (1), with significant regional differences^3^].

The ease of contagion of COVID-19 (on March 11th 2020 the World Health Organization described the Covid-19 situation as a pandemic) and the growing number of deaths (with families being decimated) along with the collapse of ICUs has prompted the authorities to adopt measures (such as generalized reduction of transport and economic activities) to prevent the virus from spreading further. These measures have caused dramatic effects (e.g., freezing of international trade, increase in unemployment, crude oil prices below zero) on the world's economy. Such effects are likely to trigger, despite Governments/Institutions' attempt to inject money into suffering economies^4^, a global recession.

In this context, biomedical experts (such as virologists, epidemiologists, immunologists, public health scholars, and statisticians) have acquired an increasingly central role in public debates. They acquired such a role by virtue of their epistemic authority (2), which loosely speaking depends on established knowledge combined with an education of excellence, success in one's field, academic achievements, recognition by colleagues, and high positions in leading institutions.

Biomedical experts have been elaborating models of contagion, strategies for preventing the virus from spreading further, and offering precious advice to politicians for implementing public health policies devised to safeguard society. In the face of a new, aggressive virus, for which there was no cure, health systems have shown themselves to be remarkably unprepared. As a consequence, the political authorities have had to rely more and more on the experts to try to formulate health policies suitable to contain the pandemic. The public too, confronted with the imminent serious threat, has not shown any of the recent tendencies of mistrust toward science and scientific reasoning recently observed (3).

Two different types of approaches to dealing with the COVID-19 pandemic have, as a result of this process, emerged. One, that is exemplified by Italy (but also shared by most governments in the world at different degrees) of severity and control, based on state-enforced quarantine. The other, exemplified by Sweden (and partly shared, at the outset at least, by countries like the USA and the UK) of relative relaxation, in which quarantine is not implemented for various reasons (economic, constitutional or alleged scientific ones) and relatively lax measures of prevention are deemed to be sufficient to stop the pandemic^5^.

In this section we briefly look at the Italian response to the coronavirus pandemic. Italy's COVID-19 epidemic, which as of July claimed more than 35,000 lives on a population of ~60 million individuals, exploded in the wealthy and prosperous North, where it put under significant pressure one of Europe's most developed health care systems.

In order to prevent mass contagion throughout the country, which would have caused catastrophic effects in the less prosperous and developed (infrastructurally, at least) South, the Italian government advised by a team of medical experts [known as *comitato tecnico scientifico*] implemented a series of measures, which involved: (i) restriction on movements; (ii) enforced quarantine; (iii) bans on travel and assemblies; (iv) closing of all stores except essential services, (v) shutting down all municipal borders; (vi) uniformed police and armed soldiers setting up checkpoints around the country.

In accord to the stringency index (which records the strictness of “lockdown style” policies that primarily restrict people's behavior) calculated by the Oxford COVID-19 Government Response Tracker^6^, at mid-March Italy scored 90.48, the most stringent level alongside with Spain. At that time Sweden scored 28.57 and it was among the countries with the least stringent measures in the world. As of mid of July, Italy scored 58.33 and Sweden 38.89.

The harsh measures implemented by the Italian government (~2 weeks after the first cases were discovered in the country's North) arguably came in too late and did not manage to prevent the surge of cases that has heavily taxed the capacity of an extremely well-regarded health care system. In particular, it is deemed that policy makers should have stressed the message “don't meet anyone” rather than merely “stay at home,” due to the special familiar and relational structure and functioning of Italian society. However, after months of lockdown, the situation in Italy was gradually getting under control and the country—as of July 2020 seemed to have “flattened the curve,” meaning that it successfully managed to slow down the spread of the infection^7^. IC units were readily available and less cases were being discovered. On these grounds, the Italian government ordered a gradual reopening of the country, even though the contagion was not zeroed.

## Sweden's Softer Approach

Sweden's COVID-19 pandemic has, as of July 2020, caused the death of almost than 6,000 people on a population of roughly 10 million individuals (Sweden's population is 1/6 of Italy). At the onset of the pandemic, the Swedish government (advised by some of the country's top epidemiologists, such as Prof Anders Tegnell) decided not to enforce lockdown (many businesses, including restaurants and bars stayed open) or to impose strict social-distancing policies (borders and schools for under-16s were also open). It only implemented a minor set of restrictions (such as banning gatherings of more than 50 people) and relatively lax trust-based measures (such as telling older people to avoid social contact or recommending work from home) to protect and safeguard society.

This was done for two reasons mostly. These are scientific/economical and constitutional. Firstly, Sweden's Public Health Agency -based on findings it gathered across the country^8^ deemed that closing-down all businesses would be useless to stop the pandemic because COVID-19 had already reached the country. In addition, the biomedical experts consulted by the government (such as Professor Anders Tegnell)^9^ remained adamant that enforced quarantine would be undesirable (for psychiatric, psychological, and physical reasons) and even counterproductive (in terms of the economic repercussions it would have on Swedish economy). Secondly, according to Swedish laws on communicable diseases^10^, it is the citizen -not the Government- that has the responsibility not to spread the disease. These laws tend to defend acquired constitutional rights (such as freedom of movement and freedom of assembly) and because of them quarantine can only be contemplated for people or small areas (such as a school or a hotel) but cannot be legally enforced on larger geographical expanses of land (e.g., regions).

Sweden's less intuitive and more controversial approach can be praised for attempting to safeguard citizens' freedom^11^, which quarantine seems to threaten. However, the potential cost in terms of human lives of this approach has also raised many concerns^12^.

Several researchers^13^ have criticized the Agency for Public Health and the experts chosen by the government for not having fully acknowledged the role of asymptomatic carriers.^14^ Others have criticized the increasingly neoliberal turn of the Swedish government, the dismantling of its health infrastructure and its large business orientation (4). Moreover, it is not clear whether this softer approach to the pandemic can really bring about the economic benefits it promises. Recent data have shown that Swedish's economy won't dodge economic hit despite its light touch to the pandemic^15^.

More importantly, despite a relatively recent study (5) suggested that Sweden's limited lockdown measures may have resulted in fewer death than expected, evidence is mounting that the Swedish's approach to curb the COVID-19 pandemic has not been as successful as first thought^16^. Mike Ryan, executive director of WHO's Health Emergencies Program, recently condemned herd immunity as a strategy to deal with the infection: “it can lead to a very brutal arithmetic that does not put people and life and suffering at the center of that equation.”^17^ Regardless of herd immunity, which clearly has not been achieved (the proportion of Swedes carrying antibodies is still believed to be well below 10%), Swedish death raise has become indeed very problematic. Sweden has a death toll greater than the United States: 564 deaths per million inhabitants compared with 444, as of July 27^18^. Sweden also has a death toll comparable to that of Italy (581)^19^ but nearly five times greater than that of the other Nordic countries combined^20^, which seems to suggest that under similar (cultural, geographical, infrastructural) conditions the death toll could have been much lower; hence, that many lives could have been saved if a different approach had been pursued.

However, as data may quickly change again, we ought to preach prudence and avoid drawing sharp conclusions. For this reason, given the evidence available at the time of writing, it seems reasonable to suggest that Swedish's approach needs -at minimum- to be redesigned, so as to take into account not just economic parameters but also to protect and defend the lives of Swedish citizens' in the interest of public health. Additionally, even if Sweden's approach would turn out to be better than the competing one (which at the moment seems very unlikely) significant concerns would remain about its possible potential application to other countries, such as Italy. Applying the Swedish approach to Italy (and to many other countries like Italy worldwide), would be quite difficult we believe, and likely result in a massacre for the following reasons. Italy's density is 206 people per Km^2^ whereas Swedish density is 1/10 of that, 25 people per Km^2^. Swedish population is, as noted above, 1/6 of the population of Italy and the number of single person households amount to ~2 million, whereas in Italy is ~8 million (on a population that is 6 times larger though). Moreover, lots of Italian towns are characterized by a rather compact layout with aggregates of houses in the city center (the architecture that make Italian towns so beautiful for tourists). Sweden, on the contrary, has many US style towns with more space between houses and families and also has a larger surface area (450,295 κM^2^ vs. Italy 301,338 κM^2^). Sweden is characterized by a high level of social and institutional trust, which is significantly lower in Italy. Finally, Swedish are on average more reserved and less outgoing than Italians, who are known to live among relatives in large communities where close contact and deep personal interactions are the social glue.

Having briefly reviewed these two approaches to the current COVID-19 pandemic, we next problematise around the epistemological stalemate that seem to arise as a consequence of their implementation in society.

## General Disagreement Among Experts: A Pressing Epistemic Problem

The two cases we discussed above are particularly instructive and offer us an opportunity to problematise about the role of science in public debates and specifically around its role in the implementation of public health policies in situations of emergency. Both these approaches are, strictly speaking, scientifically informed and epistemically justified. In brief, this seems to be a case where experts disagree, and their epistemic authority cannot be taken as the benchmark for making complex political decisions that governments should implement afterwards.

As in the case of the outbreak in the UK, scientists disagreed on herd immunity and its effectiveness as a means of controlling the spread of the SARS-CoV-2. But the key point for society was not how effective herd immunity was compared to the lockdown, but how many lives the choice of herd immunity could cost^21^.

Now, one can be an advocate of science and appreciate both the immense contribution that science has made in the constitution of our democratic States and in the solution of many daily and existential problems. Our societies certainly cannot do without science in individual lives or in the public square; however, in some cases—like the one we discussed here—the epistemic justification that underlies scientific expertise seems to be problematic and not solid enough to be uniquely used to model public health policies, which have strong normative and axiological implications for many millions of people and may affect how many lives would be spared or lost.

In this sense, both the Italian and the Swedish cases are paradigmatic examples of this problem. In Italy, the lockdown contributed to save may thousands of lives^22^, even if the human cost of the infection has been very high. Biomedical experts insisted on suggesting harsh measures of social distancing, arguing that the primary and imperative goal was to save all possible human lives. Following this approach, however, could come at the price of impoverishing the country to the point that unemployment and company closures would cause direct and indirect harms to the population not much lower than those caused by Covid-19.

In Sweden, instead, the plan agreed between biomedical experts and government was to keep the infection curve as flat as possible without blocking the country. The authorities relied on the Swedes' compliance with the rules for preventing the contagion, without direct impositions and strict sanctions. This “optimized choice” could be defended in terms of cost-benefit analysis, but it remained unclear what could be the impact of this decision in the weaker sections of society (e.g., the elderly).

In the case we present here, the lack of strong epistemic justification, which allowed for different responses to be implemented, was due to a number of reasons, the most important of which were probably (i) the novelty of the virus (previously unknown to humanity); (ii) its relatively mysterious etiology (which implied that none could really be said to be a real expert); and (iii) the fact that experts were still learning about this infection.

This means that, as we write this paper, we are in a sort of paradigm change (6), where hypotheses and theories about novel scientific facts (the COVID-19) are very fluid (hence not mature) and subject to almost immediate falsification. This stage both favors and requires consistent disagreement among experts, who sometimes - *bona fide*- even end up giving ambiguous or contradictory pieces of advice to the population (the most relevant case here being whether people should wear masks)^23^.

Part of the problem therefore seems to be epistemic in character, as it lies in the interpretation of what counts as a fact. Experts in different fields have very different beliefs about what facts are, what causes and effects are, what counts as reliable data, and indeed draw on very different sources of evidence to back their views (7).

This, again, can be easily observed in the interpretations that have formed among experts around the ways to best deal with the pandemic. On the one hand, mathematical modelers (8) assumed the virus would behave like influenza. This assumption makes people think that we may allow the virus to circulate under controlled conditions and may suggest decision makers to adopt a lax response (like the Swedish one) that tries to contain the virus spread without, for instance, harming economic activities or citizens' freedom. Other scientists and public health experts (9, 10), on the other hand, have consistently called for mass testing, tracking, and adoption of stringent measures of social isolation, which are rooted in a very different belief; the belief that the virus is not anything like common influenza and shouldn't be allowed to spread, even under controlled conditions (Italy's response).

Another part of the problem, however, is political in nature and has to do with the way certain political decisions are translated into social policies. This also relates with the topic of who chooses who and what kind of expertise is invited into those committees responsible for taking crucial decisions on public health. In the cases we have analyzed in this paper, it is clear that politics has failed to listen to society as a whole and has not used the critical tool of public reason to critically analyse and refine -when needed- the medical experts' advice.

The approach we propose here thus suggest that one informed viewpoint isn't necessarily enough or better than another informed one, but that a wider range of opinions (provided they are reasonable and sound) ought to be listened to in order for effective decision to be implemented, especially if such decisions involve normative, axiological components and are applied to public health. The idea is not just that certain expert recommendations are based on a poorly established factual basis. This is a common situation, although often overlooked.

The point is that the biomedical experts are called to advise decisions that are political in character and have enormous consequences on people's lives based on their specific scientific expertise. Such scientific expertise, in many cases, does not include public principles, values or public procedures that are instead typical of a pluralist liberal democracy. Experts typically answer technical questions and provide recommendations that are related to their expertise. Decisions with more general consequences should be made by representatives of the whole society according to formalized procedures (11–13) (Pellizzoni^24^).

## The Need of More Public Discourse in Fighting Covid-19

This means that one might call, as we do, for a broader and wider conception of expertise as well as for more representativeness, especially when scientific agreement has not crystallized yet and -like in the case we discussed above- biomedical experts alone seem unable to formulate broadly shared, uncontroversial, health policies.

For this reason, in such cases, politicians should not uncritically adopt only medical experts' opinions (which -as shown above- can be diametrically divergent); rather promote and articulate their discussions in the wider society (14), with attention to ethical and moral principles as well as to constitutional rights and to the rights of minorities (15). In brief, in light of public reason (16).

As O'Neill's brilliantly put it: “we have to supply a structure that the members of a wider, potentially diverse and unspecified, plurality can follow, by adopting and following principles of thought and action that an unrestricted audience can follow” (17). Such discussions should therefore promote a shared procedural and democratic agreement on how to act in situations of emergency (e.g., COVID-19 pandemic) with high trust being put on reliable institutions (to avoid the dangers of relativism) but also on various other forms of expertise (not only epistemic ones).

We surely welcome the recent adoption of ethical principles in many local, regional, national and international committees, especially in medicine [e.g., (18)]. We also acknowledge that, nowadays, non-biomedical experts tend to be included in many bio-medical boards and commissions. For example, bioethicists had very important roles during the Ebola epidemic (19). However, with very few exceptions (20, 21), the current COVID-19 pandemic has highlighted significant underlying epistemic ruptures between medical science, other types of expertise, the general public, and the political response. This is because bio-medical experts, by virtue of their scientific authority, have been often uncritically recognized as more authoritative than other epistemic experts or non-epistemic ones (such as human rights activists, provided that they follow some basic principle of rationality and fact verification). This is perhaps a natural assumption to make in cases like the one we discussed in this paper; however, it may lead—as we have attempted to show- to undesirable consequences and to a stalemate that may threaten the functioning of our societies. It is our opinion that the best strategy to bridge such ruptures and to avoid such problems is to open up science to public discourse and reason and include in any scientific committee responsible for taking crucial decisions on public health ethicists, bioethicists, psychologists, economists, moral and legal philosophers^25^. More importantly we believe that it may be even more fruitful to bring in and give voice to non-experts, or experts whose expertise is not based solely on “epistemic status,” but rather on either experience or political advocacy, of either the homeless, the immigrant, or other disenfranchised groups. This process may also contribute to make the epistemic expertise of experts “more representative of society as a whole.”

In order words, echoing philosopher and legal scholar Melissa Williams, we argue that “a fair and just public discourse needs at least some direct representation of the voices of those who are minorities or live in dependence because the majority groups (here experts) do not share their particular history and experience” (15).

## Conclusion

The type of expert's recommendations we have considered here, although technically flawless, are not neutral for individuals and for society and should therefore be evaluated according to procedures that do not merely assess the epistemic authority of their advocates or the adherence of their proposal to scientific criteria. The values at stake are different and often conflicting—the right to health, political freedom, the right to run a business—and the prevalence of one or the other should be entrusted to an assessment typical of decisions taken in the public sphere with the participation of various forms of expertise, chosen representatively. And just as we should never give up the contribution of (medical) experts (as in our case), so the state of emergency and the limited time available to make an effective decision, should never prevent an inclusion of normative and axiological elements in the public debate. In other words, we should be drawing on every type of potentially relevant expertise across the humanities, social and natural sciences and on insights from the wider society.

Thus, in our view, the involvement of non-biomedical experts and under-represented categories capable of drawing attention to general values, other principles and procedures should be welcomed as it could help making decisions that are more representative of society as a whole.

## Data Availability Statement

The original contributions presented in the study are included in the article/supplementary material, further inquiries can be directed to the corresponding author/s.

## Author Contributions

All authors contributed equally to the writing of this paper.

## Conflict of Interest

The authors declare that the research was conducted in the absence of any commercial or financial relationships that could be construed as a potential conflict of interest.

## Footnotes

^1^https://coronavirus.jhu.edu/#covid-19-basics (accessed August 2020).

^2^https://www.who.int/health-topics/coronavirus#tab=tab_1 (accessed August 2020).

^3^https://worldmapper.org/maps/coronavirus-cases-mortality/ (accessed August 2020).

^4^https://ec.europa.eu/info/live-work-travel-eu/health/coronavirus-response/economy_en (accessed August 2020).

^5^We are not considering here the response of some countries, like Belarus, whose President adamantly refused to accept the gravity of the pandemic and suggested its citizen to visit saunas and drink more vodka to avoid contagion: https://www.forbes.com/sites/jamesrodgerseurope/2020/04/04/in-belarus-lukashenko-has-his–own-ways-for-the-country-to-face-coronavirus/

^6^https://www.bsg.ox.ac.uk/research/research-projects/coronavirus-government-response-tracker (accessed August 2020).

^7^https://coronavirus.jhu.edu/data/new-cases (accessed August 2020).

^8^https://www.folkhalsomyndigheten.se/contentassets/1887947af0524fd8b2c6fa71e0332a87/skattning-av-vardplatsbehov-folkhalsomyndigheten.pdf?fbclid=IwAR3Dij1B7jGicxFmRtw7EODymicfo_54W0DoFz6n3Dh7ax9MSte9wnorVF4 (accessed August 2020).

^9^https://www.nature.com/articles/d41586-020-01098-x (accessed August 2020).

^10^https://www.loc.gov/law/help/health-emergencies/sweden.php (accessed August 2020).

^11^https://sverigesradio.se/sida/artikel.aspx?programid=2054&artikel=7463561 (accessed August 2020).

^12^https://www.theguardian.com/world/2020/mar/30/catastrophe-sweden-coronavirus-stoicism-lockdown-europe (accessed August 2020).

^13^A petition was launched by a group of scientists demanding the government to implement stricter measures. The petition was signed by over 2,000 doctors, including the chairman of the Nobel Foundation, Carl-Henrik Heldin.

^14^“We're not testing enough, we're not tracking, we're not isolating enough. We have let the virus loose,” Cecilia Söderberg-Naucler, an epidemiologist at the Karolinska Institute, stated. Joacim Rocklöv, a professor of epidemiology and public health at Umea University, added, “Does this mean this is a calculated consequence that the government and public health authority think is okay? How many lives are they prepared to sacrifice so as not to … risk greater impact on the economy?”: https://www.wsws.org/en/articles/2020/04/03/swed-a03.html

^15^https://www.politico.eu/article/swedens-cant-escape-economic-hit-with-covid-19-light-touch/ (accessed August 2020).

^16^https://forbetterscience.com/2020/04/07/swedish-scientists-call-for-evidence-based-policy-on-covid-19/ (accessed August 2020).

^17^https://eu.usatoday.com/story/opinion/2020/07/21/coronavirus-swedish-herd-immunity-drove-up-death-toll-column/5472100002/ (accessed August 2020).

^18^https://www.coronatracker.com/country/sweden/ (accessed August 2020).

^19^https://www.statista.com/statistics/1104709/coronavirus-deaths-worldwide-per-million-inhabitants/ (accessed August 2020).

^20^https://www.ft.com/content/46733256-5a84-4429-89e0-8cce9d4095e4 (accessed August 2020).

^21^https://www.bbc.com/news/uk-51865915 (accessed April 2020). https://www.theguardian.com/commentisfree/2020/mar/18/coronavirus-uk-expert-advice-wrong (accessed April 2020).

^22^https://spiral.imperial.ac.uk/bitstream/10044/1/77731/10/2020-03-30-COVID19-Report-13.pdf (accessed August 2020).

^23^https://www.sciencealert.com/this-is-why-advice-on-whether-you-should-wear-a-mask-is-just-so-confusing (accessed August 2020).

^24^http://www.leparoleelecose.it/?p=38050 (accessed August 2020).

^25^https://www.theguardian.com/education/2020/apr/29/to-solve-the-problems-of-this-pandemic-we-need-more-than-just-the-science (accessed August 2020).
